# Untargeted Characterization of Chestnut (*Castanea sativa* Mill.) Shell Polyphenol Extract: A Valued Bioresource for Prostate Cancer Cell Growth Inhibition

**DOI:** 10.3390/molecules25122730

**Published:** 2020-06-12

**Authors:** Nunzio Antonio Cacciola, Andrea Cerrato, Anna Laura Capriotti, Chiara Cavaliere, Maria D’Apolito, Carmela Maria Montone, Susy Piovesana, Giuseppe Squillaci, Gianfranco Peluso, Aldo Laganà

**Affiliations:** 1 Research Institute on Terrestrial Ecosystems (IRET)-UOS Naples, National Research Council of Italy, (CNR), via P. Castellino 111, 80131 Naples, Italy; nunzio.cacciola@iret.cnr.it (N.A.C.); maria.dapolito@cnr.it (M.D.); giuseppe.squillaci@iret.cnr.it (G.S.); gianfranco.peluso@cnr.it (G.P.); 2 Department of Chemistry, Università di Roma “La Sapienza”, Piazzale Aldo Moro 5, 00185 Rome, Italy; andrea.cerrato@uniroma1.it (A.C.); chiara.cavaliere@uniroma1.it (C.C.); carmelamaria.montone@uniroma1.it (C.M.M.); susy.piovesana@uniroma1.it (S.P.); aldo.lagana@uniroma1.it (A.L.); 3 CNR NANOTEC, Campus Ecotekne, University of Salento, Via Monteroni, 73100 Lecce, Italy

**Keywords:** chestnut shells, polyphenols, apoptosis, cytotoxicity, untargeted analysis, compound discoverer

## Abstract

Chestnut seeds are used for fresh consumption and for the industrial preparation of derivatives, such as chestnut flour. During industrial processing, large amounts of by-products are generally produced, such as leaves, flowers, shells and burs. In the present study, chestnut shells were extracted by boiling water in order to obtain polyphenol-rich extracts. Moreover, for the removal or non-phenolic compounds, a separation by preparative reverse phase chromatography in ten fractions was carried out. The richest fractions in terms of phenolic content were characterized by means of untargeted high-resolution mass spectrometric analysis together with a dedicated and customized data processing workflow. A total of 243 flavonoids, phenolic acids, proanthocyanidins and ellagitannins were tentatively identified in the five richest fractions. Due its high phenolic content (450.03 µg GAE per mg of fraction), one tumor cell line (DU 145) and one normal prostate epithelial cell line (PNT2) were exposed to increasing concentration of fraction 3 dry extract for 24, 48 and 72 h. Moreover, for DU 145 cell lines, increase of apoptotic cells and perturbation of cell cycle was demonstrated for the same extract. Those outcomes suggest that chestnut industrial by-products could be potentially employed as a source of bioresources.

## 1. Introduction

According to the Food and Agriculture Organization of the United Nations (FAOSTAT: http://www.fao.org/faostat/en/) data, China is the major producer of chestnut followed by southern European countries. In particular, Italy provides about 43% of the whole European chestnut production [1]. During fruit processing, several by-products are usually produced, such as leaves, flowers, shells and burs [2]. The amount of these by-products is significant, generating huge amounts of solid residues; therefore, considering the new policies developed from the European Community, it is nowadays indispensable to develop cost-efficient processing methods in order to re-use these wastes, e.g., by converting them into a valued bioresource [3]. In the case of chestnut industrial processing, an environmental problem is represented by the habit of burning the shells, a dangerous practice that may generate several toxic compounds like dioxins [4]. While, at first, shell and bur wastes were mainly used to produce fuel, they are nowadays employed to extract and recover raw material for the production of tannin extracts, which are employed for various applications, such as phenol substitutes in the formulation of wood adhesives or chrome derivatives substitutes in leather tanning [5,6,7,8].

However, in the last two decades, several studies have shown the presence of a large amount of phenolic compounds in chestnut shell with broad significant biological properties [9,10]. The vast majority of the identified phenolic compounds belong to the classes of gallotannins and ellagitannins, such as castalagin, vescalagin, acutissimin A and acutissimin B, phenolic acids, such as gallic and ellagic acids [11], and flavonoids, like catechin, catechin-gallate, quercetin and kaempferol [12]. For instance, tannins and phenolic acids were proved to show efficient antioxidant properties, specifically for lipid peroxidation inhibition [13] but also anticarcinogenic [14,15] and cardioprotective properties [6]. Moreover, phenolic compounds can prevent aging, hypertension, arteriosclerosis, and adipogenesis [10,16,17]; another study has revealed that gallic, ellagic, and syringic acids can execute anticancer effects [18]. Some of the most important flavonoids reveal good antioxidant properties, being able to activate antioxidant enzymes, reducing a-tocopherol radicals, inhibiting oxidases, mitigating nitrosative stress and increasing the levels of ureic acid and low-molecular weight compounds [19,20]. Valko et al. demonstrated that flavonoids can enhance nitric oxide status and improve endothelial function, which are important properties for the prevention of cardiovascular diseases [21]. Together with these important biological properties, the impact of chestnut by-products addition on animal diet on the microbiological, physicochemical and sensorial properties of meat products was also reported in conjunction with the evaluation of the effects of the use of chestnut by-products extracts on the quality and oxidative stability of meat products [11].

Up to now, most cited studies profiling phenolic compounds have only reported the major constituents, whereas a more detailed and comprehensive characterization completely is still lacking. With the purpose of gaining knowledge on less known compounds, high-performance liquid chromatography (HPLC) coupled to high-resolution mass spectrometry (HRMS) was chosen as the foremost technique for the comprehensive characterization of phenolic compounds. For this study, an innovative approach for the identification of phenolic compound based on HRMS data was chosen [22]. For a faster and more reliable manual validation of MS/MS spectra of known and still unknown species, an extensive database of phenolic compounds was implemented on Compound Discoverer 3.1 for the automatic match of extracted features to those present in the database. Finally, a detailed study of phenolic fragmentation pathways was achieved for the correct identification of the extracted compounds. Moreover, before mass-spectrometric analysis, a preliminary chromatographic separation of the chestnut shell extract was performed, with the purpose of eventually evaluating the richest fractions in terms of phenolic content and testing their ability to inhibit prostate cancer cell growth. In our previous study, it was determined that the crude extract derived from chestnut shells was able to inhibit the viability of different human cancer cell lines and, in particular, human prostate DU 145 cancer cell line [23]. In the present study, the cytotoxic effect of the chestnut shell phenol extract in normal epithelial cells PNT2 and prostate cancer cell line DU 145 was evaluated. Nowadays, the medicinal plants represent a never-ending source of bioactive compounds useful in the treatment of numerous disorders [24] and it has been widely accepted that their use, intended as natural product mixture, is more effective in comparison to purified compounds because of beneficial additive or synergistic interactions [25]. Thus, the complete characterization, tentative identification and bioactivity assay of the chestnut shell phenolic compounds may be the key for isolating specific molecules to use not only in cosmetic field but also in nutraceuticals and pharmaceutical fields.

## 2. Result and Discussion

In this study, chestnut shell sample extracted by hot water were characterized by means of untargeted mass spectrometric analysis and tested for potential anticancer activity studying in vitro inhibition of prostate cancer cell growth. Several studies have highlighted the presence of bioactive molecules with several biological properties in chestnut by-products that can be used in the manufacturing of health boosting-related products [1]. Thus, the recovery of bioactive compounds, mainly phenolic species, is an object of interest of industries in food, cosmetics, and energy sectors and more recently in the feed industry [11]. The nature of the solvent may significantly affect the amount of polyphenols extracted, as demonstrated in different papers in the literature [26,27].

The solvent choice fell to water since it is a bio-renewable nontoxic solvent and it could be use in large amount for a possible scale-up of analytical methodology. Therefore, chestnut shells were extracted in boiling water and lyophilized obtaining a dry extract that was characterized in terms of total phenolic content. The total phenolic content expressed as mg Gallic acid equivalents (GAE) present in one gram was 369.9 ± 7.2. With the purpose of assessing bioactivity assays in polyphenol-rich extract, a preliminary chromatographic purification and fractionation by preparative C18 RP chromatography was carried out.

### 2.1. Untargeted Characterization of Chestnut Shell Extract Fractions

**Figure 1:**
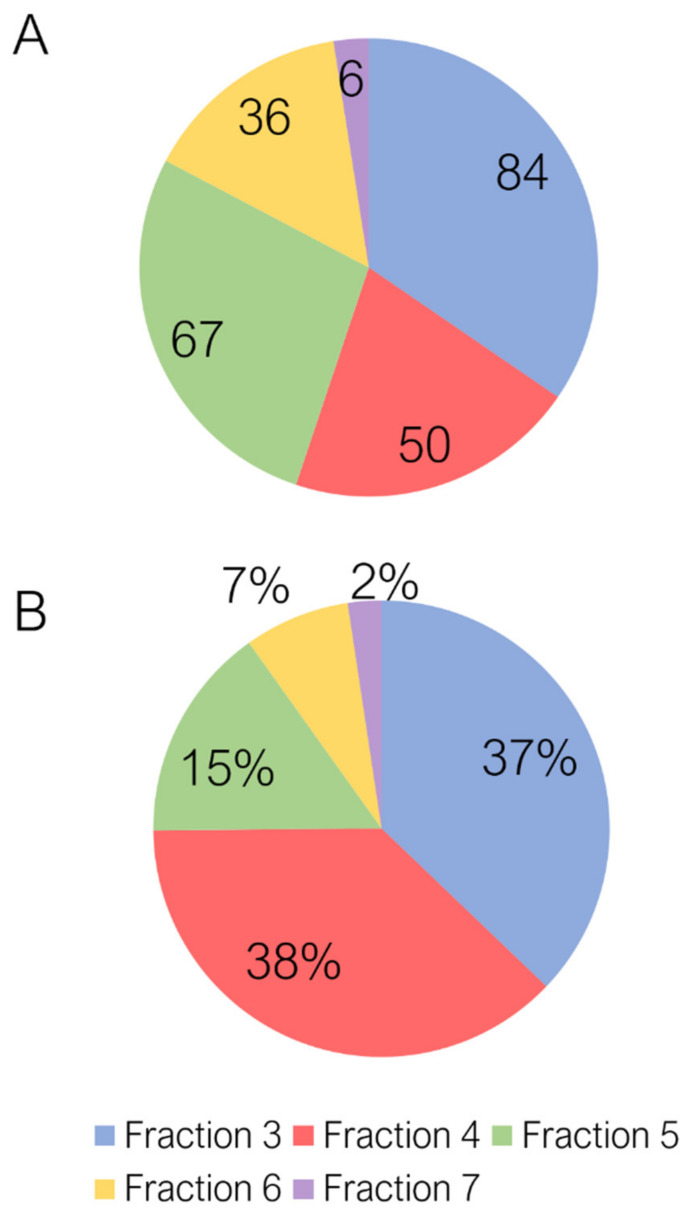
(**A**) Pie chart representing the number of tentatively identified phenolic compounds in the five analyzed fractions (F3–F7) and (**B**) the peak areas (%) of the five analyzed fractions (F3–F7) with the respect of the total peak area of tentatively identified phenolic compounds.

Ten fractions were separated by preparative RP chromatography (F1–F10). In general, the richest fractions, in terms of both numbers and areas of the tentatively identified compounds, were F3 and F4, which constituted over 75% of the total area of the identified compounds. The content in phenolic compounds progressively decreased from F4 to F7, to the extent that F7 comprises only 6 of the 243 identified phenolic (Figure 1) compounds and 2% of the total area (Figure 1). Total phenolic content of the five fractions showed that F3 presented the highest total polyphenol content (450.03 µg GAE per mg of fraction). F1 and F2 corresponded to the dead volume of the column, while F8, F9 and F10 to washing and re-equilibration and were therefore not analyzed.

With the purpose of obtaining rich fragmentation spectra for proper identification, MS^2^ spectra were acquired with three-step NCE methods in a data dependent acquisition mode untargeted analysis in both positive and negative polarity [22]. Moreover, the study of retention times of free and conjugated compounds was used to confirm the identity, since the latter are supposed to be eluted earlier from the C_18_ column due to the polar nature of sugars. For spectral extraction, alignment and analysis, a dedicated and customized data processing workflow on Compound Discoverer 3.1 was employed. Briefly, a database was compiled and implemented in the software by means of Excel, including 23,596 free and conjugated flavonoids, 19,305 free and conjugated phenolic acids and 2645 proanthocyanidins and ellagitannins. Free flavonoids and phenolic acids, termed aglycones, were included, as well as glycosylated forms with one, two or three sugar moieties. In addition, compound class scoring tool was enabled by including a list of characteristic fragment ions. By applying filters based on exact mass and match to fragments, a large screening of the identified features was accomplished. This automatic screening was followed by the manual study and validation of the MS^2^ spectra, to assign a confidence level to each identification [22]. In order to evaluate the content of non-phenolic species, a general metabolomics workflow analysis was also performed by match of MS^2^ spectra to online available databases.

In F3, proanthocyanidins, and particularly polymers of catechin and gallocatechin, are the most abundant class of phenolic compounds both in terms of numbers and areas (46%) (Figure S1). Among the identified proanthocyanidins, the most abundant species are dimers of catechin (procyanidin B2), dimers of gallocatechin (prodelphinidins) and mixed dimers of catechin and gallocatechin. As regards ellagitannins, low-molecular weight derivatives of gallic acid (several isomers of digalloyl hexose) were identified, as well as some more structurally complex compounds (castalagin, pedunculagin and geraniin isomers). The tentatively identified flavonoids are all flavanol derivatives, such as glycosyl and acyl derivatives of (epi)catechin and (epi)gallocatechin, and several low-molecular weight and extremely hydrophilic free, acylated and glycosylated phenolic acids were identified. Non-phenolic compounds constituted only 6% of the total area. Detailed data for tentatively identified compounds are reported in Supplementary Materials Table S1.

In F4, more than 60% of the total area is represented by the two epimers of catechin, which are also the most abundant compound identified in the chestnut shell extract (Figure S2). Regarding the polymeric species, several higher molecular weight derivatives of gallic acid were identified (trigalloyl hexose and galloyl hexahydroxydiphenoyl hexose isomers) as well as some malabathrin and mongolicain isomers. Moreover, several a and b-linked type proanthocyanidin dimers, trimers and tetramers were identified. As in fraction 3, non-phenolic compounds do not contribute significantly to the composition of the fraction.

F5 was the most heterogeneous in its composition, even though monomeric phenolics are significantly more abundant then polymeric species (Figure S3). In contrast to F3 and F4, other classes of flavonoids rather than flavanols are present, such as flavonols (myricetin derivatives), O-methyl flavonols (laricitrin derivatives), flavanones (naringenin derivatives) and flavanonols (dihydromyricetin and taxifolin derivatives). Among the identified phenolic acids, free ellagic acid is the most abundant, as well as free and acylated hydroxybenzoic and hydroxycinnamic acids. High-molecular weight derivatives of gallic and hexahydroxydiphenic acid (HHDP) comprised the class of ellagitannins (such as tetra- and pentagalloyl-hexose isomers), as well as heavy complex species, such as rugosin, mongolicain, malabathrin and punicalagin. Several a-linked type proanthocyanidins were also identified. Those species are, in fact, generally more hydrophobic than the common b-linked type, due to the conjugation of one of the hydroxyl groups.

In F6 and F7, polymeric polyphenols were almost completely absent, being generally more hydrophilic than monomeric compounds. Phenolic acids represent about 14% of the total area of F6 and, among these, free or non-glycosylated derivatives of hydroxycinnamic acids, such as caffeic and ferulic acid, were the most abundant (Figure S4), However, flavonoids, and specifically glycosylated and acyilated derivatives of O-methyl flavonols (isorhamnetin, laricitrin and syringetin), dominated the composition of this fraction, representing almost 60% of the total area.

The phenolics content of F7 is almost negligible with respect to other classes of phytochemicals (Figure S5). Among the few phenolic compounds, some free flavonoids, such as quercetin and laricitrin, were identified. Nearly 80% of the total area of the tentatively identified compounds in fraction 7 is represented by other phytochemicals, like terpenes and terpenoids. It is worth mentioning that such species are generally poorly analyzed in liquid chromatography, therefore, supposedly, there were many other species which were not properly identified in this fraction. Moreover, in Supplementary Materials, trends of the main classes of phenolic compound in the five analyzed fractions are also discussed.

**Figure 2:**
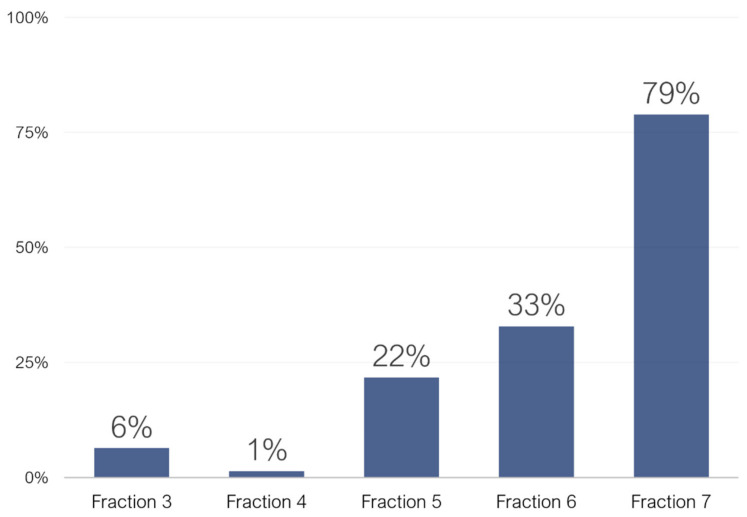
Bar chart displaying the peak areas (%) of non-phenolic compound with respect of the total peak area of tentatively identified compounds in fractions F3–F7.

Finally, from general metabolomics analysis of the five fractions (F3–F7), the content of non-phenolic species was evaluated. While non-phenolic compounds areas were negligible for F3 and F4, their value progressively increased for the other fractions (Figure 2). Those identified compounds belonged mainly to the classes of terpenes, terpenoids, carboxylic acids and aromatic species. As far as the evaluation of the bioactivities was concerned, F3 and F4 were therefore considered the best options. As the content of phenolic compounds was overwhelmingly higher than other species, in fact, any found bioactivity could be more easily assigned to phenolic compounds.

### 2.2. Fraction 3 Treatment Decreased Cell Viability in a Time- and Dose-Dependent Manner

**Figure 3:**
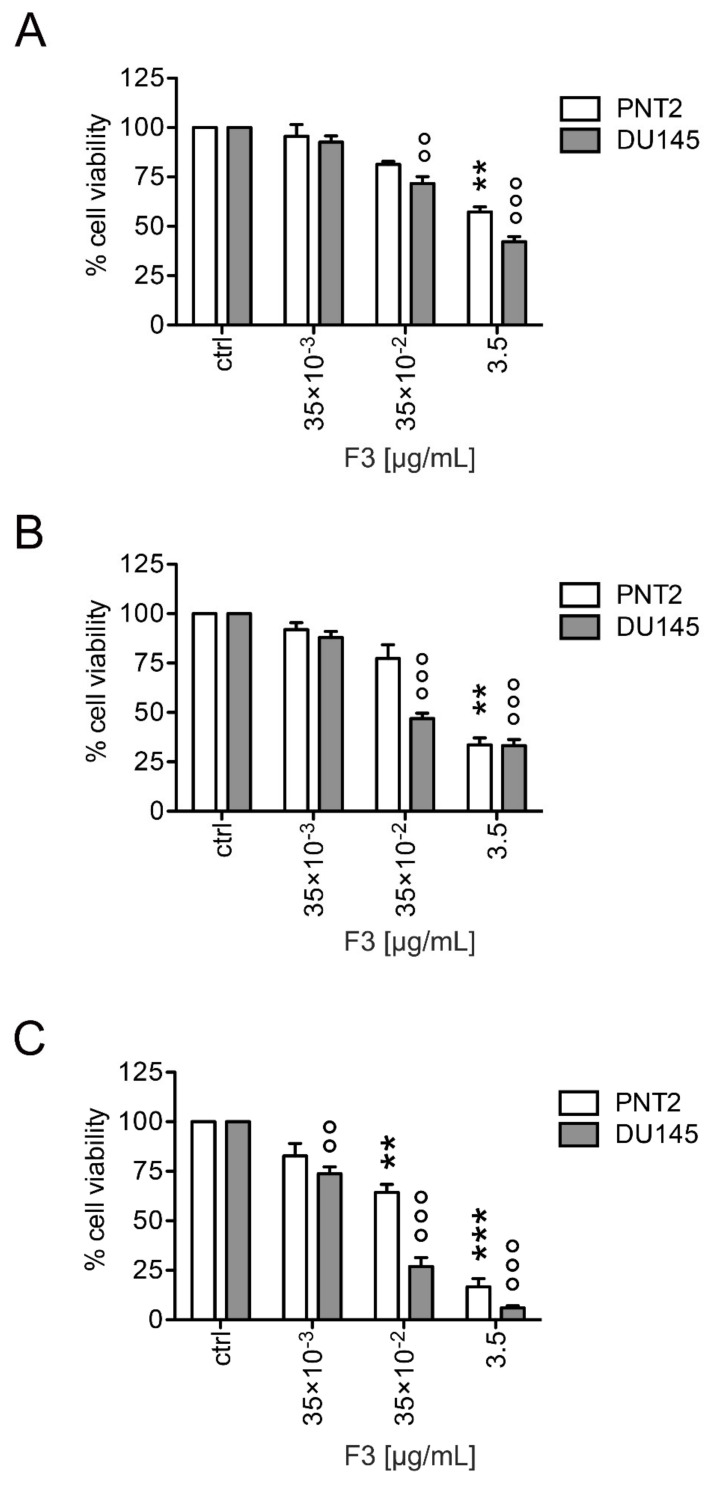
Inhibitory effect of F3 on PNT2 and DU 145 cell viability. The cells were treated with different concentrations (35 × 10^−3^, 35 × 10^−2^ and 3.5 μg/mL) of F3 for 24- (**A**), 48- (**B**), and 72 (**C**) h. Data are expressed as mean ± SD of three independent experiments ** (*p* < 0.01); *** (*p* < 0.001) F3-treated PNT2 vs. untreated PNT2 (control); °° (*p* < 0.01); °°° (*p* < 0.001) F3-treated DU 145 vs. untreated DU 145 (control).

To investigate the cytotoxic activity of F3 against prostate cells, we treated both DU 145 and PNT2 cells with different F3 concentrations (0–3.5 µg/mL) for 24, 48, and 72 h (Figure 3). Cell viability was assessed by using the Trypan blue assay. As depicted in Figure 3, F3 treatment a reduction of the cell viability in both DU 145 and PNT2 prostate cell lines in a time- and concentration-dependent manner. A significant cytotoxic effect of F3 treatment at was evident in DU 145 cells at all the time points evaluated. On the contrary, the viability of F3-treated PNT2 cells at 35 × 10^−2^ concentration decreased significantly only after 72 h incubation time, but remained, however, much higher than that of the corresponding DU 145 cells. A different sensitivity to the F3 cytotoxic effect was also confirmed through IC_50_ determination. Indeed, DU 145 cells exhibited approximately 18-fold higher sensitivity (IC_50_ = 0.08 ± 0.13 µg/mL) to F3 compared to PNT2 cells (IC_50_ = 1.43 ± 0.21 µg/mL). Based on these results, further experiments to evaluate biological effects of the extracts were carried-out using 35 × 10^−2^ μg/mL F3 concentration.

### 2.3. Fraction 3 Treatment-Induced Cell Death in DU 145 Cells Occurs Through Apoptosis

**Figure 4:**
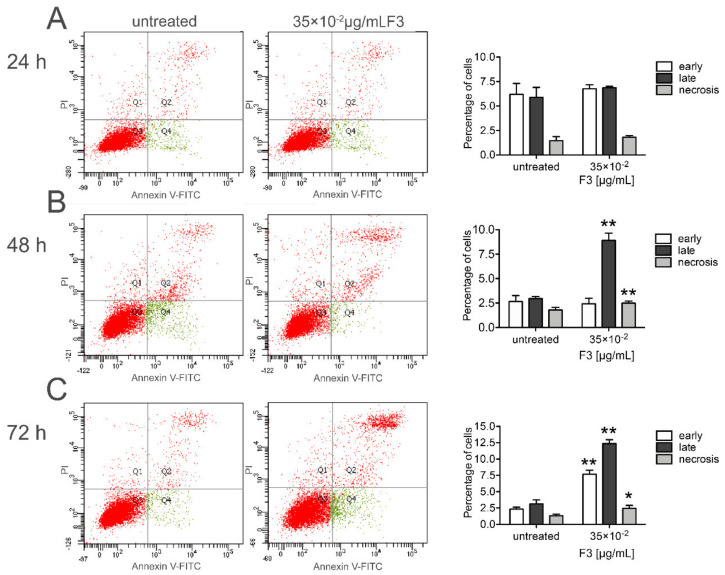
F3 treatment-induced apoptosis in DU 145 cells. The cells were exposed to 35 × 10^−2^ μg/mL F3 for 24- (**A**), 48- (**B**) and 72 (**C**) h and apoptosis was analyzed by flow cytometry. The results are expressed as mean ± SD of three independent experiments * (*p* < 0.05), ** (*p* < 0.01).

To examine whether the inhibitory activity of F3 was caused by cell apoptosis or necrosis, we analyzed DU 145-cell death by flow cytometry. Annexin V staining was employed to establish the percentage of apoptotic/necrotic cells induced by F3 after 24, 48, and 72 h of treatment (Figure 4). In the quadrant dot plots, the lower left indicates viable cells (Annexin V^−^/PI^−^), the lower right, early apoptotic cells (Annexin V^+^/PI^−^), the upper right, late apoptotic cells (Annexin^+^/PI^+^) and the upper left, necrotic cells (Annexin^−^, PI^+^). As shown in Figure 4A, the exposure of DU 145 cells to 35 × 10^−2^ μg/mL F3 did not cause a significant change in the early/late apoptotic nor necrotic stage cell populations. Conversely, DU 145 cells treated with F3 for 48 h showed an increase in both late (8.90 ± 0.76% vs. 2.9 ± 0.21% in untreated cells) apoptotic- and necrotic- (2.50 ± 0.20% vs. 1.80 ± 0.26% in untreated cells) stage populations (Figure 4B). Finally, at 72 h of treatment, the exposure of DU 145 cells to F3 treatment further determined an increase in both early- (7.67 ± 0.61% vs. 2.33 ± 0.31% in untreated cells) and late- (12.37 ± 0.61% vs. 3.13 ± 0.61% in untreated cells) apoptotic stage population along with a concomitant increase (2.46 ± 0.45 % vs. 1.33 ± 0.25% in untreated cells) also in necrotic stage population (Figure 4C). On the contrary, a very slight increase in late apoptotic stage population (~1.8-fold increase) was observed in PNT2 cells treated with 35 × 10^−2^ μg/mL F3 concentration.

### 2.4. Fraction 3 Treatment Induced a Perturbation of DU145 Cell Cycle

**Figure 5:**
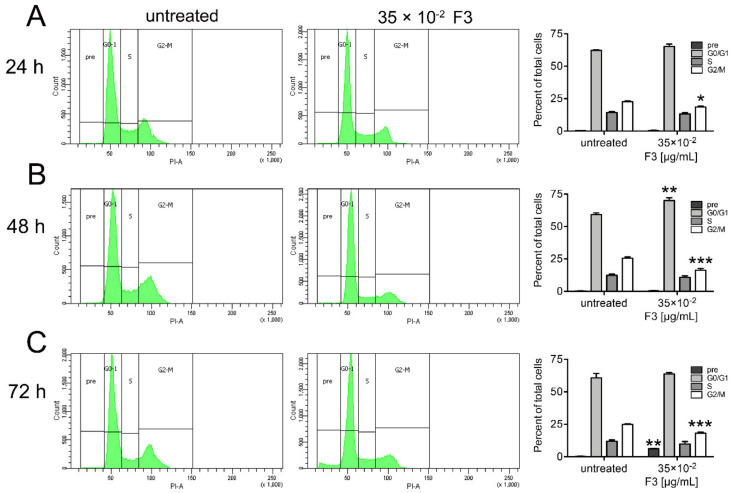
Effect of F3 on cell cycle arrest regulation in DU 145 cells. The cells were exposed to 35 × 10^−2^ μg/mL F3 for 24- (**A**), 48- (**B**) and 72 (**C**) h and cell cycle distribution was analyzed by flow cytometry (FCM). The results are expressed as mean ± SD of three independent experiments * (*p* < 0.05), ** (*p* < 0.01), *** (*p* < 0.001).

DNA fragmentation and damage represent the major hallmarks that signal the cells to undergo apoptosis. To better establish whether the apoptotic cell death induced by F3 also affects cell cycle distribution, we examined the cell cycle in DU 145 cells upon exposure to 35 × 10^−2^ μg/mL CDSE-3 for 24, 48, and 72 h (Figure 5). Cell cycle analysis was carried-out by using flow cytometry following staining of the cells with PI, a fluorescent DNA-intercalating molecule. As shown in Figure 5A, after 24 h exposure, F3 caused a significant accumulation of the cells at G2/M phase (18.60 ± 0.60% vs. 22.6 ± 0.67% in untreated cells). Subsequently, following 48 h F3 treatment (Figure 5B), we also found that percentage of G0/G1 phase cells was increased (70.03 ± 2.05% vs. 59.20 ± 1.32% in untreated cells) with a reduced percentage of G2/M phase cells (16.33 ± 1.33% vs. 25.57 ± 0.99% in untreated cells), accordingly. At 72 h point, F3 treatment led to a significant accumulation in sub-diploid DNA, a feature of apoptotic cells, that increased in the sub-G1 position of the cell cycle (6.2 ± 0.30% vs. 0.3 ± 0.20% in untreated cells), with a low percentage of G2/M phase cells (18.13 ± 0.83% vs. 24.90 ± 0.56% in untreated cells), respectively (Figure 5C).

In our previous study, we assessed the anticancer activity of the crude chestnut shell extract and found that it showed promising anti-proliferative and pro-apoptotic activities against different cancer cell lines [23]. In the present study, due to its high abundance of phenolic compounds, the effects of F3 in human cancer cells were investigated. Therefore, the antiproliferative and pro-apoptotic activity as well as the cell cycle inhibition of this fraction in DU 145 cells used as in vitro model were evaluated. The increased antiproliferative activity might be due to the concomitant presence of proanthocyanidins (e.g., Procyanidin B2) and gallocathechins dimers (e.g., prodelphinidin) as well as to the presence of complex ellagitannins (Castalagin, pedunuclagin and geraniin) found in F3. As far as proanthocyanidins and gallocatechins dimers are concerned, our results are in agreement with a previous work in which prodelphinidins extracted from fresh green tea leaves were able to exert antiproliferative and cytotoxic activities, induction of apoptosis as well as inhibition of cell cycle progression at G0/G1 cell cycle phase in human A549 non-small lung cancer cells [28]. Additionally, our results are also in line with those of Santulli C et al. in which Castalagin contained in *Castanea sativa* bark extract contributed to the apoptosis induction and cell cycle arrest in SH-SY5Y neuroblastoma cell lines, respectively [29]. It is worth mentioning that in the study of Kuo PL et al., after 48 h of treatment, the maximal effect on proliferation inhibition was observed with 20 µM prodelphinidin which inhibited proliferation in 63.2% of A549 cells [28]. In our study the effect of F3 on DU 145 cell viability was more marked (>50%) probably due to the cooperation of different compounds acting via synergistic or additive manner. It is crucial for a plant extract to display high cytotoxicity against cancer cells, with little effect against normal cells. F3 fits this feature, as DU 145 cells exhibited a higher sensitivity to F3 treatment compared to PNT2 prostate normal cells. This observation agrees with our previous work in which chestnut shell extracts, after 24 h of treatment, also showed a reduced cell viability in DU 145 in comparison to normal epithelial prostate cells PNT2 [23]. This study also pointed out that the phenolic compounds present in F3 contributed to the apoptosis induction in DU 145 cells since the number of early- and late-apoptosis as well as necrotic cells increased after 72 h of F3 treatment suggesting a late apoptosis signal that could reflect an “apoptosis-necrosis continuum” [30]. These results are also in line with those of Agarwal C. et al in which grape seed proanthocyanidin extract induced apoptosis in human prostate cancer cells DU 145 without affecting the growth and viability of the normal cells [31]. The results also revealed that F3 caused cell cycle inhibition in DU 145 at G0/G1 checkpoint after 48 h of treatment, in agreement with the findings of Kuo et al. in which authors demonstrated that Prodelphinidin B2 at 10 µM concentration increased the population of G1 phase from 34.7 to 42.3% whereas the same cell treated with 20 µM of prodelphinidin B-2 increased the number of G1for up to 60.9% [28].

## 3. Material and Methods

### 3.1. Chemicals and Reagents

The chestnut shell waste was kindly provided by a food factory located near Avellino (Italy). By-product material was mashed, freeze-dried and stored at −20 °C until use. Optima^®^ LC-MS grade water, methanol (MeOH) and acetonitrile (ACN) were purchased from Thermo Fisher Scientific (Waltham, MA, USA).

Reagents used for the biological tests were obtained from suppliers as follows: Rosewell Park Memorial Institute 1640 medium (RPMI 1640) and Dulbecco’s Modified Eagle Medium (DMEM), foetal bovine serum (FBS), penicillin/streptomycin solution, L-glutamine, 0.25% trypsin-EDTA and phosphate-buffed saline (PBS) were purchased from Gibco by Life Technologies (Grand Island, NY, USA). Chemicals for total phenolic content determination (Folin–Ciocalteu reagent and Na_2_CO_3_), bovine serum albumin (BSA) for total proteins estimation, and reagents for reducing sugars determination (3,5-dinitrosalicylic acid-DNS, sodium hydroxide and sodium potassium tartrate), gallic acid and glucose were purchased from Sigma-Aldrich Co. (Milano, Italy).

### 3.2. Extract Preparation from Chestnut Shells

Chestnut shells were dried in oven at 55 °C until reaching constant weight, then ground using a food homogenizer (type 8557-54, Tefal, France). The bioactive molecules were extracted as follows: chestnut shells (5% *w*/*v*) were suspended in deionized water, and boiled for 1 h under continuous stirring. The mixture was cooled on ice and centrifuged at 3220× *g* for 1 h at 4 °C (Eppendorf 5810R). After recovering the supernatant, the solid residue was rinsed with the same volume of water lost during the boiling procedure. Then, the mixture was centrifuged as above and the supernatant was added to the previous one in order to restore the original volume. The obtained solution (extract) was lyophilized in an Edwards Modulyo freeze-dryer (Edwards, Cinisello Balsamo, Milano, Italy), and the powder (dry extract-DE) was stored at room temperature.

### 3.3. Total Phenolic Content

A stock solution of 3 mg DE/mL was prepared in phosphate-buffered saline (PBS) for analyses. All tests were carried-out in triplicate and results were expressed as mean ± Standard Deviation (SD).

Total phenolic content was measured by the Folin–Ciocalteu method [32]. Aliquots of the chestnut shell extract, diluted to 150 μL with phosphate-buffered saline (PBS), were mixed with 750 μL of Folin-Ciocalteu reagent (diluted ten-folds with deionized water) and 600 μL of 7.5% (*w*/*v*) Na_2_CO_3_. The reaction was developed in the dark at 25 °C for 2 h, and the absorbance was read at 765 nm against a blank prepared with 150 μL of PBS (Varian Cary 100 Scan, Varian Analytical Instruments, Torino, Italy). The total phenolic content was estimated by a calibration curve prepared with increasing quantities of gallic acid standard solution (1.5–10 μg). The results were expressed as mg GAE (Gallic Acid Equivalents)/g DE.

### 3.4. Purification of Phenolic Compounds by HPLC

Extracted phenolic compounds were purified using an Xbridge BEH preparative C_18_ 5 μm OBD 10 × 250 mm (Waters) connected to Shimadzu Prominence LC-20A system, including a CBM-20A controller, two LC-20 AP preparative pumps, a DGU-20A3R online degasser. An SPD-20A UV with a preparative cell (0.5 mm) was used as detector. A FRC-10A Shimadzu was employed as the autocollector. Data acquisition was performed by the LabSolution version 5.53 software (Shimadzu, Kyoto, Japan). The detector was set at 320 nm. Samples were eluted with a flow rate of 7 mL min^−1^ using ddH_2_O/TFA (99.9/0.1, *v*/*v*) as mobile phase A and MeOH/TFA (99.9/0.1, *v*/*v*) as mobile phase B. The gradient started from 0% B and then increased to 60% in 25 min; then, B was increased to 100% and maintained constant for 4 min. The column was re-equilibrated for 2 min. Ten fractions were collected every 3 minutes, except for fraction 1 and 2 (dead volume) and 9 and 10 (washing and column equilibration). Collected fractions (F3–8) were analyzed by UHPLC coupled to high resolution mass spectrometry.

### 3.5. UHPLC-MS/MS Analysis

A Vanquish binary pump H (Thermo Fisher Scientific, Bremen, Germany), equipped with thermostated autosampler and column compartment, was used for polyphenol chromatographic separation on a Kinetex core-shell C_18_ column (100 × 2.1 mm i.d.) with particle size of 2.6 µm (Phenomenex, Torrance, CA, USA) at 40 °C and with a flow-rate of 600 µL min^−1^. The elution gradient and mobile phases were optimized in a previous work [33].

The chromatographic system was coupled to a Q Exactive hybrid quadrupole-Orbitrap mass spectrometer (Thermo Fisher Scientific) with a heated ESI source. The ESI source parameters were set as reported in our previous works [22,34].

For both low and high-molecular weight polyphenols, detection was conducted in TOP 5 DDA acquisition mode, consisting in a first full-scan acquisition, followed by the fragmentation of the five most intense ions detected in full-scan mode. An exclusion list containing the most intense ions detected in a blank sample, consisting of H_2_O/MeOH (90:10, *v*/*v*), was added to the mass-spectrometric method.

For low-molecular weight polyphenol analysis (flavonoids and phenolic acids) and for high-molecular weight polyphenol analysis (tannins), MS data were acquired in the range 150–1000 *m*/*z* and 300–2000 *m*/*z*, respectively, with a resolution (full width at half maximum, FWHM, at *m*/*z* 200) of 70,000. In full scan mode, the automatic gain control (AGC) target value was 200,000 and the maximum ion injection time was 100 ms. The isolation window width was 2 *m*/*z*. MS^2^ fragmentation was performed with a resolution (FWHM, at *m*/*z* 200) of 35,000 with AGC target value set at 100,000 and dynamic exclusion set to 3 s. A stepped collision energy (CE) fragmentation was achieved in the HCD cell at three values of normalized collision energy (NCE), namely, 20–50–80 NCE in positive mode and 20–40–60 NCE in negative mode.

All samples were run in triplicate followed by the injection of the standard mix and a blank sample of H_2_O/MeOH (90:10, *v*/*v*). The injection volume was 10 µL.

### 3.6. Data Analysis and Phenolic Compound Validation

For each fraction, raw data obtained from three consecutive injections and from the blank sample were processed by Compound Discoverer using a customized method [22]. For both low- and high-molecular weight polyphenols raw data processing, customized databases generated by the combination of free phenolic compounds with several sugars, aliphatic and aromatic acids and complete of IDs, masses and molecular formulas, were implemented in the mass list feature for the automatic matching of extracted *m*/*z* ratios. Moreover, detailed HCD fragmentation spectra for flavonoids and phenolic acids were implemented in compound class scoring section for automatic MS^2^ spectra matching and parameters for predict composition tool were adapted to polyphenol analysis.

Extracted masses from the chromatograms were filtered to remove background compounds found in the blank sample, compounds whose *m*/*z* ratios could not derive from masses present in the databases and those which were not fragmented. Furthermore, for flavonoid and phenolic acid data processing, compounds whose compound class scores were 0% for all compound classes, were also filtered out. Finally, filtered compounds were manually validated by matching fragmentation spectra to those of available standards or to spectra reported in the literature. When data were lacking, phenolic compounds were tentatively identified according to the characteristic fragmentation spectra.

### 3.7. Cell Cultures

The following cell lines were used in this study: human prostate cancer cells DU 145 (ATCC^®^ HTB-81) and human immortalized non-cancerous prostate epithelial cells PNT2 (ECACC 95012613). The cells were purchased from the American type culture collection (ATCC, Manassas, VA, USA) and European Collection of Cell Cultures (ECACC, Salisbury, UK). PNT2 cells were cultured in RPMI 1640 medium, while DU 145 cells were grown in DMEM medium. All the cells were supplemented with 10% fetal bovine serum (FBS), 1% L-glutamine, 50 U/mL penicillin, 50 mg/mL streptomycin maintained at 37 °C in a 95% air and 5% CO_2_ atmosphere.

### 3.8. Assessment of Cell Viability by Trypan Blue Assay

To determine whether the CDSE fractions 3 (F3) was able to cause perturbation in cell viability; the Trypan blue assay was used. In brief, both the cell lines were seeded onto flat-bottom 6-well plates at a density of 1.5 × 10^5^ cells/well in culture media containing 10% FBS. After 24 h of incubation, cells were treated with increasing concentrations (35 × 10^−3^, 35 × 10^−2^ and 3.5 μg/mL) of F3 in a medium supplemented with 1% FBS for 24–72 h. Control cells were treated with the vehicle (PBS) used for dissolving the fraction. After treatment, the cells were resuspended in trypan blue solution and counted using a digital cell counter instrument (Biorad TC20, Hercules, CA, USA). The numbers of viable cells in control cell culture without F3 was compared with the number of viable cells in CDSE-3 treated culture. IC_50_ value was determined by counting the cells after trypan blue staining using vehicle treated cells as control after 48 h incubation time. The experiment was repeated a minimum of three independent times and data was presented with standard deviation (SD).

### 3.9. Evaluation of Apoptosis by Flow Cytometry (FCM)

DU 145 cells were seeded in 6-well plates at a density of 1.5 × 10^5^ cells and allowed to adhere for 24 h. Then, the cells were treated with F3 in medium containing 1% FBS for 24–72 h. Subsequently, the apoptosis was evaluated by FCM using Annexin V/fluorescein isothiocyanate (FITC) and propidium iodide (PI) apoptosis assay (Dojindo Molecular Technologies Inc., Munich, Germany), as already described [23]. Briefly, cells were suspended in Annexin-binding buffer and incubated with 5 μL of Annexin V-FITC and 5 μL of PI for 15 min at room temperature and analyzed with FACSCanto II (BD Biosciences, MI, Italy) by DIVA software. For each condition, at least 20,000 events were recorded.

### 3.10. Cell Cycle Analysis by FCM

DU 145 cells were seeded in 100 mm dishes at density of 7 × 10^5^ cells and maintained in culture for 24 h. Subsequently, the cells were serum-starved for 18 h to synchronize them. Then, the cells were treated with F3 in medium containing 1% FBS for 24–72 h. DU 145 control and treated-cells were trypsinized and fixed at 4 °C in 70% ice-cold ethanol. After fixation, the cells were washed with PBS and stained with 20 μg/mL PI in presence of 0.1 mg/mL RNase A for 1 h a 37 °C. The stained cells were analyzed with FACSCanto II (BD Biosciences, MI, Italy) by using the DIVA software.

### 3.11. Statistical Analysis

Statistical analysis for the cytotoxicity, apoptosis and cell cycle assays were carried-out by using GraphPad Prism 5.01 software (La Jolla, San Diego, CA, USA). Results were expressed as mean-standard deviation (SD) of three independent experiments. Student’s *t*-test was used for comparison between groups. Results with *p* < 0.05 were considered statistically significant.

## 4. Conclusions

In conclusion, untargeted mass-spectrometric analysis together with dedicated tools for tentative identification of phenolic compounds has shown promising results in terms of comprehensive characterization of valuable plant by-products. Bioactive compounds extracted from waste matrices could be of incredibly high industrial interest for nutraceutical and pharmaceutical applications. Our results confirm that chestnut shell extracts have the potential to be better chemopreventive agents compared to isolated compounds, as the minor constituents along with major components present in plant extracts work in synergy and thus provide significantly better results [15].

## Supplementary Materials

Table S1: list of tentatively identified phenolic compounds in F3 of chestnut shell extract; Figure S1: composition of F3 in terms of tentative identifications and peak areas for the four main classes of phenolic compounds, Figure S2: composition of F4 in terms of tentative identifications and peak areas for the four main classes of phenolic compounds; Figure S3: composition of F5 in terms of tentative identifications and peak areas for the four main classes of phenolic compounds¸ Figure S4: composition of F6 in terms of tentative identifications and peak areas for the four main classes of phenolic compound; Figure S5: composition of F7 in terms peak areas for the four main classes of phenolic compounds; Figure S6: bar chart summarizing trends in the total peak areas of the four main classes of phenolic compounds in the 5 analyzed fractions; Figure S7: bar chart summarizing the trends of ellagitanins and proanthocyanidins in the 5 analyzed fractions in terms of total peak areas; Figure S8: bar chart summarizing the trends of phenolic acids in the 5 analyzed fractions in terms of total peak areas; Figure S9: bar chart summarizing the trends of flavonoids in the 5 analyzed fractions in terms of total peak areas; Figure S10: bar chart summarizing the trends of flavan and flavone derivatives in the 5 analyzed fractions in terms of relative abundance.

## References

[1] Squillaci G., Apone F., Sena L.M., Carola A., Tito A., Bimonte M., De Lucia A., Colucci G., La Cara F., Morana A. (2018). Chestnut (*Castanea sativa* Mill.) industrial wastes as a valued bioresource for the production of active ingredients. Process. Biochem..

[2] Pinto D., Braga N., Silva A.M., Costa P., Delerue-Matos C., Rodrigues F., Galanakis C.M. (2019). Chestnut. Valorization of Fruit Processing By-Products.

[3] Piovesana S., Capriotti A.L., Cavaliere C., La Barbera G., Montone C.M., Chiozzi R.Z., Lagana A. (2018). Recent trends and analytical challenges in plant bioactive peptide separation, identification and validation. Anal. Bioanal. Chem..

[4] Liberti A., Goretti G., Russo M.V. (1983). PCDD and PCDF formation in the combustion of vegetable wastes. Chemosphere.

[5] Vazquez G., Freire M.S., Santos J., Antorrena G., González-Álvarez J. (2010). Optimisation of Polyphenols Extraction from Chestnut Shell by Response Surface Methodology. Waste Biomass Valoriz..

[6] Braga N., Rodrigues F., Oliveira M.B.P.P. (2014). *Castanea sativa* by-products: A review on added value and sustainable application. Nat. Prod. Res..

[7] Vazquez G., González-Álvarez J., Santos J., Freire M.S., Antorrena G. (2009). Evaluation of potential applications for chestnut (*Castanea sativa*) shell and eucalyptus (*Eucalyptus globulus*) bark extracts. Ind. Crop. Prod..

[8] Comandini P., Lerma-García M.J., Simó-Alfonso E.F., Toschi T.G. (2014). Tannin analysis of chestnut bark samples (*Castanea sativa* Mill.) by HPLC-DAD–MS. Food Chem..

[9] Vella F.M., Laratta B., La Cara F., Morana A. (2017). Recovery of bioactive molecules from chestnut (*Castanea sativa* Mill.) by-products through extraction by different solvents. Nat. Prod. Res..

[10] Youn U.-Y., Shon M.-S., Kim G.-N., Katagiri R., Harata K., Ishida Y., Lee S.-C. (2016). Antioxidant and anti-adipogenic activities of chestnut (*Castanea crenata*) byproducts. Food Sci. Biotechnol..

[11] Echegaray N., Gómez B., Barba F.J., Franco D., Estévez M., Carballo J., Marszałek K., Lorenzo J.M. (2018). Chestnuts and by-products as source of natural antioxidants in meat and meat products: A review. Trends Food Sci. Technol..

[12] Barreira J.C., Ferreira I.C.F.R., Oliveira M.B.P.P., Pereira J.A. (2010). Antioxidant Potential of Chestnut (*Castanea sativa* L.) and Almond (*Prunus dulcis* L.) By-products. Food Sci. Technol. Int..

[13] Barreira J.C., Ferreira I.C.F.R., Oliveira M., Pereira J.A., Oliveira M.B.P.P. (2008). Antioxidant activities of the extracts from chestnut flower, leaf, skins and fruit. Food Chem..

[14] Lee J.H., Khor T.O., Shu L., Su Z.-Y., Fuentes F., Kong A.-N. (2012). Dietary phytochemicals and cancer prevention: Nrf2 signaling, epigenetics, and cell death mechanisms in blocking cancer initiation and progression. Pharmacol. Ther..

[15] Dashwood R.H. (2007). Frontiers in Polyphenols and Cancer Prevention. J. Nutr..

[16] Rasouli H., Farzaei M.H., Khodarahmi R., Farzei M.H. (2017). Polyphenols and their benefits: A review. Int. J. Food Prop..

[17] Vauzour D., Rodriguez-Mateos A., Corona G., Concha M.J.O., Spencer J.P. (2010). Polyphenols and Human Health: Prevention of Disease and Mechanisms of Action. Nutrients.

[18] Sorice A., Siano F., Capone F., Guerriero E., Picariello G., Budillon A., Ciliberto G., Paolucci M., Costantini S., Volpe M.G. (2016). Potential Anticancer Effects of Polyphenols from Chestnut Shell Extracts: Modulation of Cell Growth, and Cytokinomic and Metabolomic Profiles. Molecules.

[19] Prochazkova K., Boušová I., Wilhelmova N. (2011). Antioxidant and prooxidant properties of flavonoids. Fitoterapia.

[20] Sroka Z. (2005). Antioxidative and Antiradical Properties of Plant Phenolics. Zeitschrift für Naturforschung C.

[21] Valko M., Leibfritz D., Moncol J., Cronin M.T., Mazúr M., Telser J. (2007). Free radicals and antioxidants in normal physiological functions and human disease. Int. J. Biochem. Cell Boil..

[22] Cerrato A., Cannazza G., Capriotti A.L., Citti C., La Barbera G., Laganà A., Montone C.M., Piovesana S., Cavaliere C. (2020). A new software-assisted analytical workflow based on high-resolution mass spectrometry for the systematic study of phenolic compounds in complex matrices. Talanta.

[23] Cacciola N.A., Squillaci G., D’Apolito M., Petillo O., Veraldi F., LaCara F., Peluso G., Margarucci S., Morana A. (2019). *Castanea sativa* Mill. Shells Aqueous Extract Exhibits Anticancer Properties Inducing Cytotoxic and Pro-Apoptotic Effects. Molecules.

[24] Veeresham C. (2012). Natural products derived from plants as a source of drugs. J. Adv. Pharm. Technol. Res..

[25] Caesar L.K., Cech N.B. (2019). Synergy and antagonism in natural product extracts: When 1 + 1 does not equal 2. Nat. Prod. Rep..

[26] Gironi F., Piemonte V. (2011). Temperature and solvent effects on polyphenol extraction process from chestnut tree wood. Chem. Eng. Res. Des..

[27] Fernández-Agulló A., Freire M.S., Antorrena G., Pereira J.A., González-Álvarez J. (2014). Effect of the Extraction Technique and Operational Conditions on the Recovery of Bioactive Compounds from Chestnut (*Castanea sativa*) Bur and Shell. Sep. Sci. Technol..

[28] Kuo P., Hsu Y., Lin T., Lin C. (2005). The antiproliferative activity of prodelphinidin B-2 3′-O-gallate from green tea leaf is through cell cycle arrest and Fas-mediated apoptotic pathway in A549 cells. Food Chem. Toxicol..

[29] Santulli C., Brizi C., Durante M., Micucci M., Budriesi R., Chiarini A., Frosini M. (2018). Apoptotic-induced Effects of *Castanea sativa* Bark Extract in Human SH-SY5Y Neuroblastoma Cells. Nat. Prod. Commun..

[30] Elmore S.A. (2007). Apoptosis: A Review of Programmed Cell Death. Toxicol. Pathol..

[31] Agarwal C., Singh R.P., Agarwal R. (2002). Grape seed extract induces apoptotic death of human prostate carcinoma DU145 cells via caspases activation accompanied by dissipation of mitochondrial membrane potential and cytochrome c release. Carcinogenesis.

[32] Singleton V.L., Rossi J.A. (1965). Colorimetry of Total Phenolics with Phosphomolybdic-Phosphotungstic Acid Reagents. Am. J. Enol. Vitic..

[33] La Barbera G., Capriotti A.L., Cavaliere C., Piovesana S., Samperi R., Chiozzi R.Z., Lagana A. (2017). Comprehensive polyphenol profiling of a strawberry extract (*Fragaria* × *ananassa*) by ultra-high-performance liquid chromatography coupled with high-resolution mass spectrometry. Anal. Bioanal. Chem..

[34] Cavaliere C., Antonelli M., Capriotti A.L., La Barbera G., Montone C.M., Piovesana S., Laganà A. (2019). A Triple Quadrupole and a Hybrid Quadrupole Orbitrap Mass Spectrometer in Comparison for Polyphenol Quantitation. J. Agric. Food Chem..

